# Silk‐based conductive materials for smart biointerfaces

**DOI:** 10.1002/SMMD.20230004

**Published:** 2023-04-17

**Authors:** Fanfan Fu, Dongmei Liu, Yilun Wu

**Affiliations:** ^1^ School of Environmental and Biological Engineering Nanjing University of Science and Technology Nanjing China; ^2^ School of Materials Science and Engineering Nanyang Technological University Singapore Singapore; ^3^ School of Computer Science and Engineering Nanjing University of Science and Technology Nanjing China; ^4^ College of Biotechnology and Pharmaceutical Engineering Nanjing Tech University Nanjing China; ^5^ School of Biological Sciences Nanyang Technological University Singapore Singapore

**Keywords:** biointerface sensor, biomedical scaffold, electronic conductivity, regenerated silk fibrion, soft electronics

## Abstract

Silk‐based conductive materials are widely used in biointerface applications, such as artificial epidermal sensors, soft and implantable bioelectronics, and tissue/cell scaffolds. Such biointerface materials require coordinated physicochemical, biological, and mechanical properties to meet current practical needs and future sophisticated demands. However, it remains a challenge to formulate silk‐based advanced materials with high electrical conductivity, good biocompatibility, mechanical robustness, and in some cases, tissue adhesion ability without compromising other physicochemical properties. In this review, we highlight recent progress in the development of functional conductive silk‐based advanced materials with different morphologies. Then, we reviewed the advanced paradigms of these silk materials applied as wearable flexible sensors, implantable electronics, and tissue/cell engineering with perspectives on the application challenges. Silk‐based conductive materials can serve as promising building blocks for biomedical devices in personalized healthcare and other fields of bioengineering.

## Graphical abstract

In this review, we highlight recent progress in the development of functional conductive silk‐based advanced materials with different morphologies. We, then, discuss the practical applications and challenges faced by silk‐based advanced materials in the areas of biomedical interface applications, such as wearable flexible sensors, implantable electronics, and tissue/cell engineering.

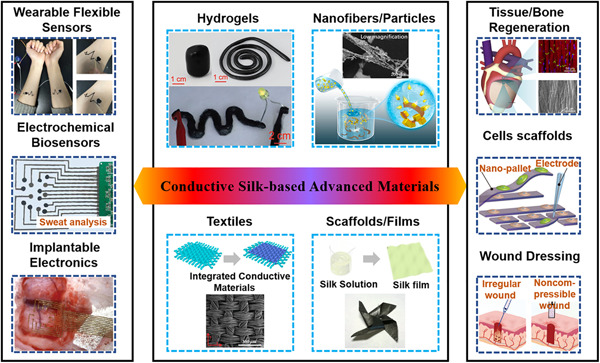

1

**Key points**

- Diverse forms of smart silk‐based materials are introduced.
- The applications of conductive silk‐based smart materials in biological interfaces are presented.
- Future directions and challenges of conductive silk materials in next‐generation biomedical devices are presented.

## 2 INTRODUCTION

Biointerface materials combined with biological tissues/cells can provide controllable mediators for information exchange and transfer biomolecules between abiotic and living organisms, which have aroused extensive research interest in the fields of bioelectronics, diagnostics, and advanced therapies.^1^, ^2^, ^3^, ^4^ Owing to their fascinating physicochemical properties, biointerface materials have been employed in realms of wearable/implantable electronics, electrochemical biosensors, and tissue/cell engineering.^5^, ^6^, ^7^, ^8^ For these biomaterials, the main features are good biocompatibility, and in some cases, biodegradability, followed by comfort, flexibility, and mechanical robustness for integrating with biological tissue. Currently, the mainstream of biointerface materials, taking the field of bioelectronics as an example, is still based on inorganic materials with appropriate mechanical robustness and electrical conductivity, such as silicon and metals.^9^, ^10^ However, such inorganic materials are limited by the incoordination of their mechanical/chemical properties with biological tissues, which may lead to some serious problems, such as unreliable signal acquisition, nonconforming contact, and in vivo inflammatory responses. Since these problems are difficult to overcome by only using inorganic‐based materials, breakthroughs would be made by incorporating organic biomaterials, especially natural polymers.^11^, ^12^, ^13^, ^14^, ^15^

Silk (from *Bombyx mori*), as a representative of natural biopolymers, is composed of a silk fibroin (SF) (70–80 wt%) core with a layer of sericin shell coating. Due to their inherent merits in physicochemical, biological, and mechanical properties, silk‐based materials satisfy the requirements of biointerface materials in suitable biocompatibility, biodegradability, ultrahigh strength, and flexibility.^16^, ^17^, ^18^, ^19^ Generally, silk fibroin with good biocompatibility is accessible through the regenerated silk fibroin (RSF) process by removing sericin after degumming raw silk fibers in an alkaline solution. The silk fibroin is composed of nano‐*β*‐crystallites (hydrogen bonding) linked by amorphous peptide chains, which can play a dominant role in the regulation of mechanical properties of RSF. The amorphous peptide chain provides elasticity in silk fiber, while the *β*‐crystalline domain could impart desirable mechanical strength and stiffness. In addition, the abundant functional groups (e.g. primary amino group) on RSF peptide potentiate chemical modification and adhesion to tissues/organs. Especially, such functional groups facilitate their biomodification/integration with other functional materials, such as conductive polymer/nanofillers, endowing the intrinsically insulated silk fibroin polymers with electronic properties.

**FIGURE 1:**
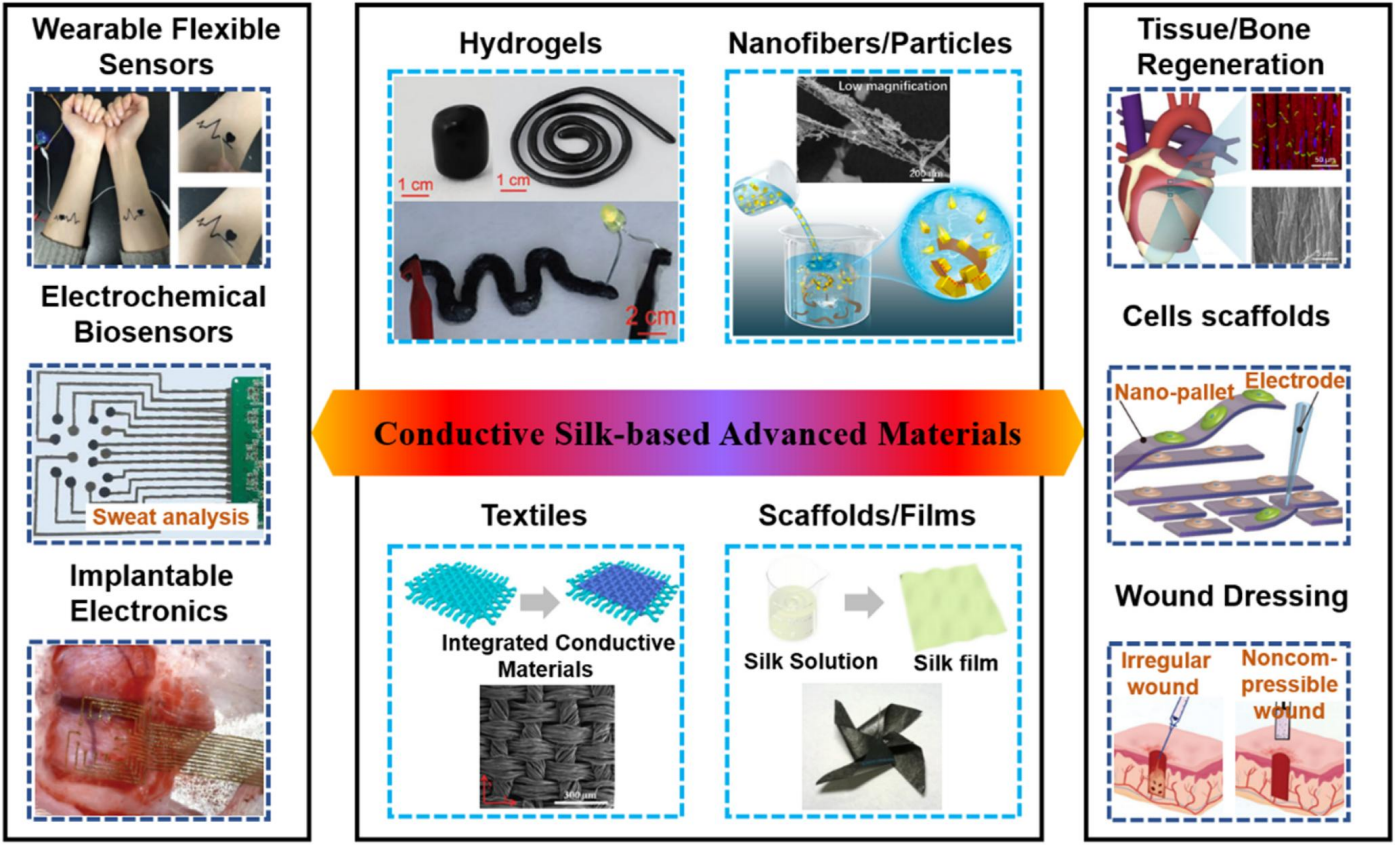
Conductive silk‐based advanced material for biomedical applications. The left column shows the conductive silk‐based materials used for wearable flexible sensors,^24^ electrochemical biosensors,^23^ and implantable electronics,^25^ respectively (Reprinted with permission. Copyright 2019, John Wiley and Sons; Copyright 2019, The Authors, published by AAAS; Copyright 2020, Elsevier). The middle column summarizes main forms of conductive silk‐based materials ^26^, ^27^, ^28^, ^29^ (Reprinted with permission. Copyright 2021, John Wiley and Sons; Copyright 2019, American Chemical Society; Copyright 2016, John Wiley and Sons; Copyright 2014, The Royal Society of Chemistry). The right column presents the application of conductive silk‐based materials in the areas of tissue/bone regeneration,^30^ cell scaffold,^31^ and wound dressing,^32^ respectively (Reprinted with permission. Copyright 2022, Elsevier; Copyright 2016, John Wiley and Sons; Copyright 2022, John Wiley and Sons).

Among those functional silk variants, the conductive silk‐based advanced materials have attracted much attention.^20^, ^21^ When designing conductive silk materials, the presence of active functional sites in their structures facilitates their incorporation with various conducting elements, such as conductive polymers, carbon‐based fillers, and metallic interfaces, resulting in highly conductive silk fibroin complexes.^22^ On the other hand, the silk can also be transformed into carbon materials with intrinsic nitrogen doping and can exhibit extraordinary electrical conductivity.^23^ Therefore, conductive silk‐based materials could be engineered into various forms, such as hydrogels, fibers, films, scaffolds, and powders, ranging from nano, micro, to macro dimensions. Owing to excellent properties in electronic conductivity, combined with controllable forms, high mechanical robustness, programmable biodegradation, and non‐inflammatory degradation products, the conductive silk advanced materials have promising potential in biomedical applications, such as wearable flexible sensors, electrochemical biosensors, implantable electronics, tissue/bone regeneration, cells scaffold, and wound dressing (Figure 1).

Conductive silk‐based materials for biomedical interfaces often require coordinated physicochemical, biological, and mechanical properties to meet the practical application requirements.^33^, ^34^ High electrical conductivity, good biocompatibility, mechanical robustness, and (in some cases) tissue adhesion capabilities are the basic requirements for most biomedical applications. For conductive polymer‐based silk composites, their high electronic conductivity is orchestrated by the π‐bond structure of conducting polymers, while this structure also endows the composites with relatively fragile mechanical strength. For the conductive filler‐based silk composites, high electronic conductivity usually requires a high content of conductive fillers considering the insulation of silk protein, while a high infill rate of conductive fillers easily induces phase separation of the composites, resulting in poor mechanical properties. In addition, conductive silk‐based materials composed of mobile charges, such as ionic liquids, salt ions, and metal ions, also face problems, such as poor electrical conductivity and interface biocompatibility. Therefore, to develop conductive silk‐based advanced materials for biointerface applications, the main challenge lies in achieving high electronic conductivity with uncompromised physicochemical properties.

Generally, the main forms of conductive silk materials for biomedical applications are hydrogel, nanofibers/particles, textiles, and films/scaffolds. In this review, we first discuss the preparation approaches and technics of different forms of silk protein‐based materials, followed by the construction methods for the formation of conductive pathways. Furthermore, recent advances in conductive silk‐based materials for various biointerface applications are highlighted, followed by a discussion of obstacles and remaining challenges in this field. This review aims to provide useful insights and guidelines for the development and design of conductive silk advanced materials for biointerface applications.

## 3 FORMS OF SILK‐BASED MATERIALS

Developing multiple formats of silk‐based materials is also critical to the success of biological applications. By tuning the chemical composition and the physical structure of silk molecules, and combining with the different processing techniques, silk‐based materials with various formats can be prepared, such as hydrogel, scaffold (films and 3D porous aerogels), nanofibers/particles, and textiles.^35^ Most of these silk forms are typically derived from the fibroin solution (organic or aqueous), which can be chemically activated with other functional materials and shaped by micromachining technology. In this section, we focus on the current processing and preparation methods of various forms of silk‐based advanced materials and emphasize the impact of chemical and physical structure switching to the conductivity of silk materials.

### 3.1 Hydrogels

**FIGURE 2:**
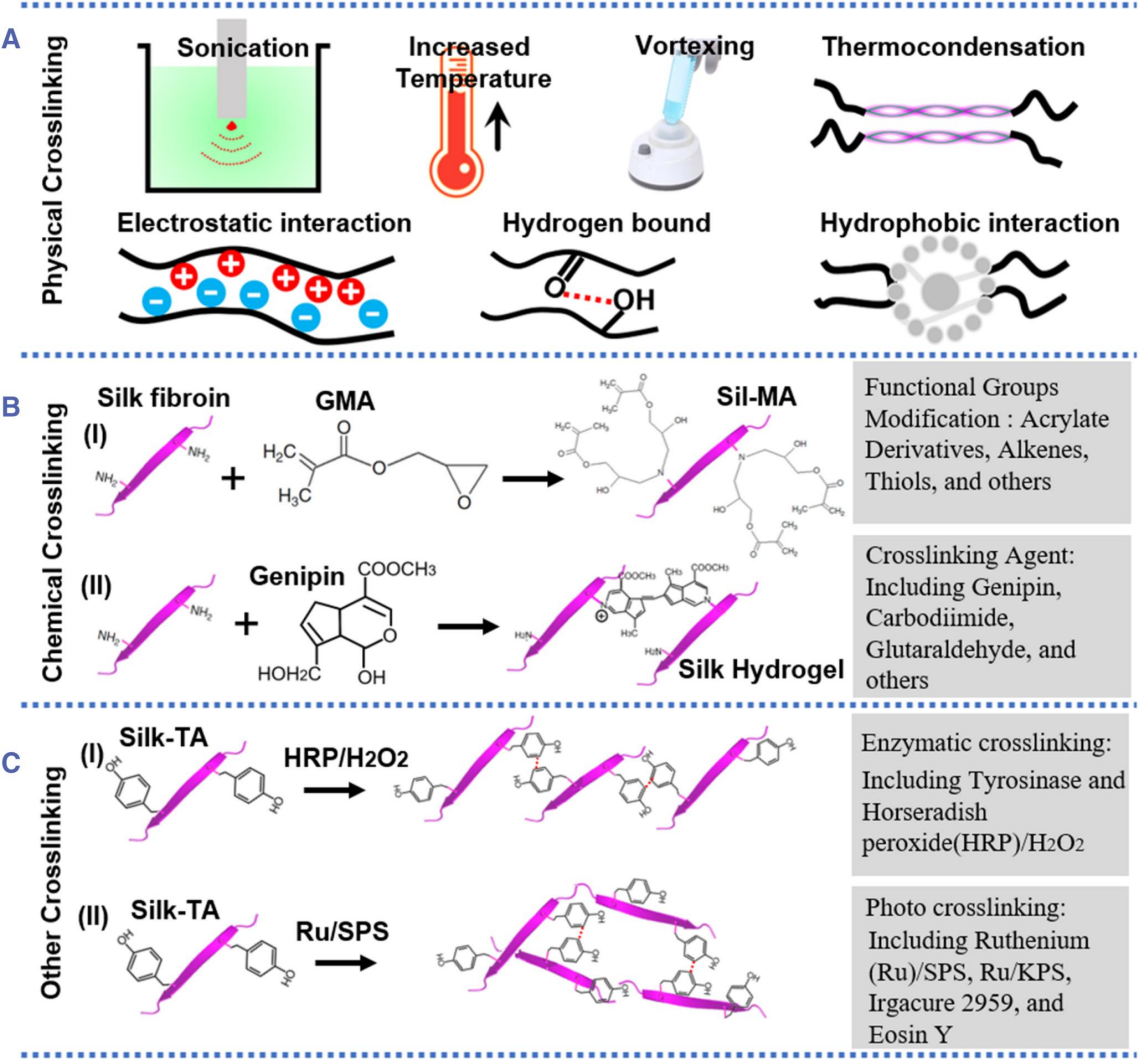
Cross‐linking methods to form silk SF hydrogels. (A) Physical cross‐linking: SF hydrogel prepared by forming the molecular entanglement or molecular interaction under physical conditions, such as hydrogen bound, electrostatic, and hydrophobic interaction. (B) Chemical cross‐linking: SF hydrogel prepared by modifying functional groups on the SF chain, such as acrylate derivatives, alkenes, and thiols. (C) Enzymatic/photo cross‐linking: SF hydrogel networks formed by enzymes or photo initiation, such as in peroxide (HRP)/H_2_O_2_ and ruthenium (Ru)/sodium persulfate (SPS) systems.

Silk‐based hydrogels have emerged as promising biological platforms and have been widely used in cell/drug carriers, extracellular matrix‐like (ECM‐like) scaffolds, and tissue fillers.^36^, ^37^, ^38^ According to the hydrogel network formation conditions, the common cross‐linking methods of silk‐based hydrogel include physical cross‐linking, chemical cross‐linking, and enzyme‐based or photo‐based methods (cataloged as other crosslinking) as shown in Figure 2. Theoretically, physical cross‐linked silk hydrogels are usually fabricated by accelerating molecular motions using heating, robust vortexing, or strong sonication (Figure 2A). Such active intermolecular movement in a silk solution could lead to the foster interactions of hydrophobic domains and form *β*‐sheet cross‐linked points. Other non‐covalent interactions, such as hydrogen bonding, van der Waals forces, and electrostatic interactions, are also physical cross‐linking mechanisms for silk‐based hydrogel preparation. The physically cross‐linked silk hydrogels have significant advantages, such as simple processing and good biocompatibility without any chemical additives, making them promising for biological applications, such as minimally invasive procedures and injection. However, these hydrogels face poor mechanical properties as these physical interactions are reversible and relatively weak in force, especially for those systems with abundant *β*‐sheet domains.^39^, ^40^

Silk hydrogels based on the chemical cross‐linking method illustrate improved mechanical properties with the help of irreversible covalent bonds. The chemical cross‐linking hydrogels mainly rely on the modification of functional groups in SF molecules or the use of functional small molecules as cross‐linking agents to form chemical cross‐linking points between the SF molecules. The primary amines and carboxylic acid groups of SF are accessible targets for SF molecular modification.^41^ As shown in Figure 2B, the functional groups (such as Alkenes, Thiols, Acrylate derivatives, etc.) were grafting on SF molecules to construct chemical cross‐linking points, and cross‐linking agents (such as genipin, carbodiimide, glutaradehyde, etc.) were also used to link SF molecules for the synthesis of a hydrogel network. In addition, the SF hydrogel based on chemical bonds can also be compounded with other functional polymers to form a complex hydrogel system, such as the double network hydrogel, which can construct an energy dissipation mechanism and greatly enhance mechanical properties of the hydrogel. These chemically cross‐linked SF hydrogels do not fully exploit the self‐assembly process of silk fibroin, making them different from many other biopolymer‐based hydrogels. The additional chemicals and new chemical bonds introduced into the hydrogel system may bring extra concerns, such as the degradation of biomolecules and the biocompatibility of the whole hydrogel.

Many other cross‐linking methods have been also used to control the SF solution to a hydrogel transition, including but not limited to enzymatic crosslinking, photo crosslinking, and electric crosslinking, as shown in Figure 2C.^42^ These cross‐linking methods are simple and versatile by using light, enzymes, or electricity as initiators. In addition, these hydrogels are formed by a spontaneous assembly or an enzymatic chain reaction and are not affected by other chemical reagents and processing techniques. The physical and chemical properties of hydrogels are designable according to actual application requirements, regardless of the impact on biological activity. For example, multifunctional silk hydrogels with homogenous structures were designed to improve cell compatibility.^43^ Overall, since the physicochemical properties of hydrogels are closest to those of human tissues and organs, the silk‐based hydrogel with multiple functions will be further studied and improved.

### 3.2 Scaffolds

**FIGURE 3:**
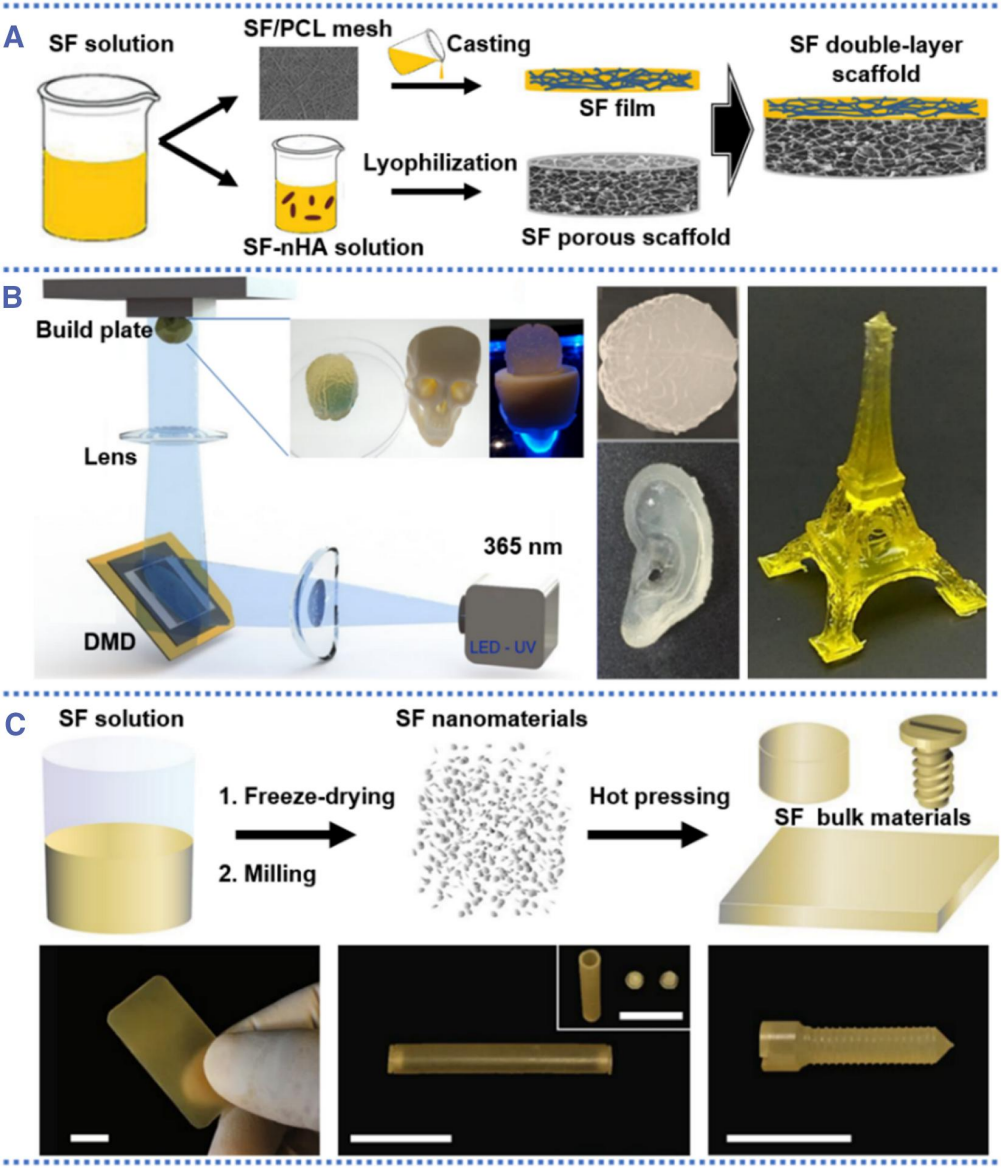
Silk scaffold derived from different processing methods. (A) Schematic diagram of a double‐layer scaffold based on the SF film and porous sponges^44^ (Reprinted with permission. Copyright 2022, Taylor & Francis). The SF film was prepared by casting the SF solution into SF/PCL mesh membrane, and the porous sponges were obtained by freeze‐drying SF solutions. SF scaffold with different morphologies from (B) digital light printing^45^ and (C) the thermoplastic molding method.^46^ Scale bars are 1 cm in (C) (Reprinted under terms of the CC‐BY license. Copyright 2018, The Authors, published by Springer Nature. Reprinted with permission. Copyright 2020, The Authors, published by Springer Nature).

Silk‐based advanced materials in the form of 3D scaffolds are particularly prominent in the fields of tissue engineering and cell culture due to their good biocompatibility, low immunogenicity, and controllable biodegradability. These scaffolds can be made into different shapes by using pure silk materials or doping with other biopolymer/natural proteins. Apart from biological and chemical properties, the scaffolds possess unique morphology characteristics, such as their topology, hierarchy, and pore microstructures. These morphology characteristics are critical for the practical application, especially for thin film‐like and porous sponge‐like scaffolds. As shown in Figure 3A, a double‐layer scaffold based on the SF film and porous sponges was fabricated from SF solutions through a specific drying process.^44^ Generally, with the volatilization of the aqueous solution or organic solvent, the SF solution spin coated or vertically deposited on different substrates will be transformed to a thin film state with the help of chemical bonds or physical interaction between the silk protein molecules. On the other hand, for the fabrication of porous sponge scaffolds, a conventional manufacturing method is freeze‐drying technology. By controlling the SF concentration, pH, and freezing temperature, the pore structure within the sponge scaffold, such as pore size and porosity, can be efficiently tuned.^47^, ^48^ Besides freeze‐drying technology, salt leaching and gas foaming techniques are other methods to prepare porous sponge scaffolds.^49^ For example, in a typical experiment, the sponge scaffolds for cell culture could be fabricated by adding porogens, such as sodium chloride particulates, to a silk solution (hexafluoroisopropanol, HFIP). The pore structure of these sponge scaffolds depends on the size, homogeneity, and distribution of the porogens.^50^

3D printing technology also provides an effective and controllable method for producing silk‐based scaffolds. In contrast to these conventional techniques, 3D printing can construct complex structures layer by layer in accordance with computer‐aided design.^51^, ^52^ For example, the digital light processing 3D bioprinting was used to create highly complex scaffolds, such as the brain and ear organ structures, as well as the Eiffel Tower with a small grid and holes on the surface, as illustrated in Figure 3B.^45^ Such silk‐based scaffolds could be applied to tissue and organ engineering in accordance with the unique needs of biology. On the other hand, silk is limited by the *β*‐sheet nanocrystallites, which could degrade before melting when exposed to thermal processing. This imposes restrictions on the fabrication of silk‐based scaffolds using solution‐derived processing methods, such as silk protein stability and solubility in the solution, as well as cost and time efficiencies. Therefore, David L. Kaplan et al. presented a thermal processing technique for direct solid‐state molding of regenerated silk into bulk scaffolds.^46^ The scaffolds obtained by the thermoplastic molding method can also be shaped into different morphologies with tunable mechanical properties (Figure 3C).

### 3.3 Fibers

**FIGURE 4:**
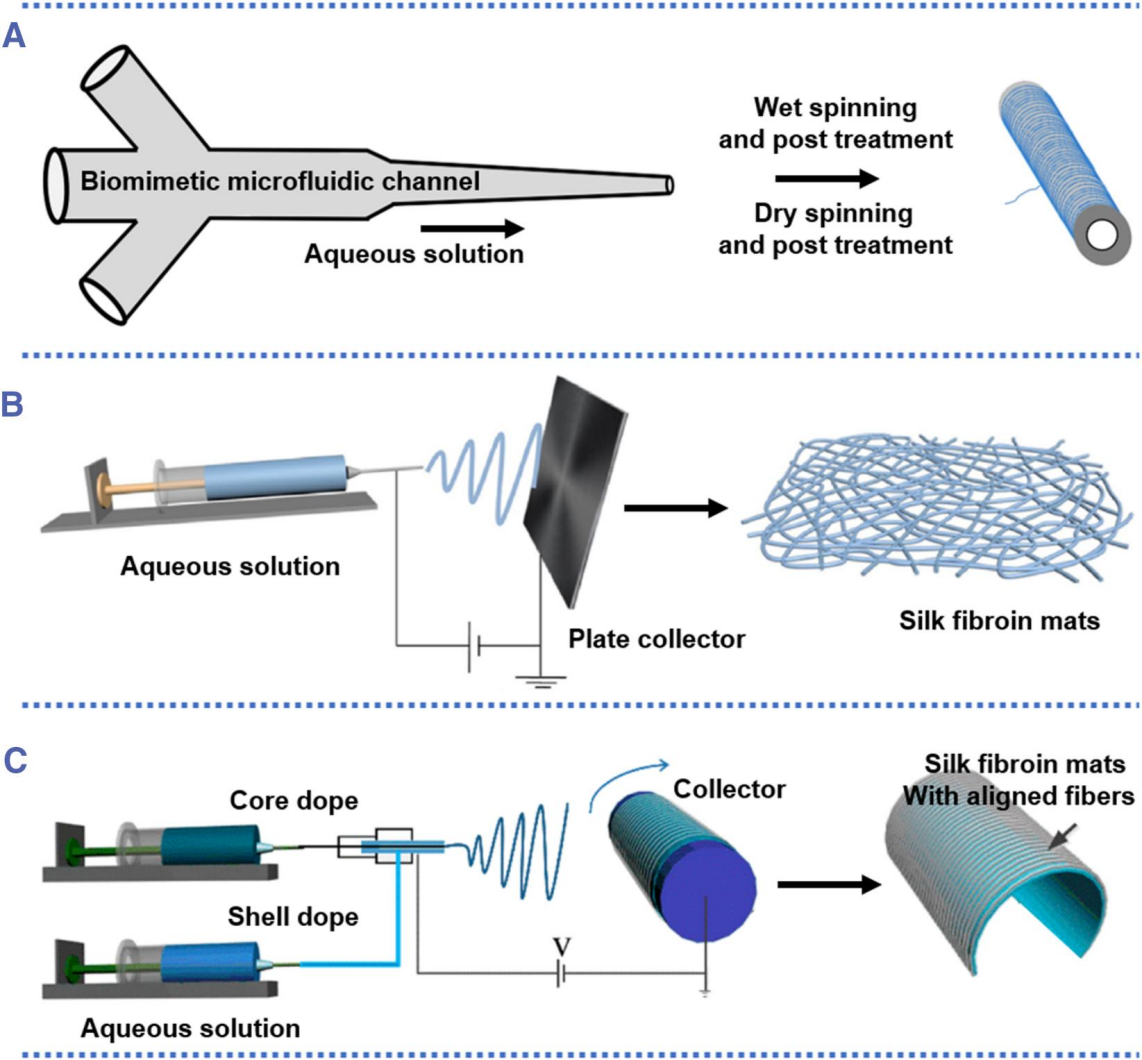
SF fibers from several processing methods. (A) Schematic diagram of SF fibers fabricated by the microfluidic chip, (B) electrospinning with the plate/mesh collector, and (C) coaxial electrospinning with the round roller collector^55^ (Reprinted with permission. Copyright 2022, The Authors, published by Elsevier).

Most of the silk fibroin appeared as a fiber form in nature. Understanding the natural silk spinning process benefits the development of novel procedures to regenerate silk fibers.^53^, ^54^ Inspired by nature, different types of regenerated silk fibers were designed and manufactured by spinning technology for a particular need (Figure 4).^56^ Wet spinning and dry spinning are the most simple and cost‐effective ways to process silk fiber. In contrast to dry spinning, which extrudes a silk fibroin stock solution into air for drying, wet spinning employs a syringe to extrude the solution into a coagulation bath (such as alcohol, ferric, and polyethylene glycol [PEG]‐based solutions).^57^ Both wet and dry spinning can be combined with microfluidic chips,^58^, ^59^, ^60^ which facilitate the control of various conditions, such as shear forces, ion concentration, and pH gradients (Figure 4A). The efficient combination with microfluidic chips enables the mimic to the actual biological process in silkworm, leading to the creation of silk fiber with desired functions.^61^ For example, Buehler et al. demonstrated a bioinspired method for spinning regenerated silk fibers by employing SF that were only partially dissolved as the spinning dope. By controlling the shear/stress elongation of dope during the spinning process, the formed regenerated silk fiber could feature with hierarchical and polymorphic structures and exhibit robust mechanical properties.^62^

On the other hand, electrospinning is an effective method to produce silk fibers/mats with nanometer scale diameters. During the electrospinning, the increased voltage between the silk fibroin solution droplets and the collector enables the droplets to overcome surface tension and eventually form a jet stream. The solvent of the jet stream will be evaporated on the way, forming the SF fibers on a plate collector (Figure 4B). The form of collectors can be diversified (a stationary/high‐speed rotating flat surface, drum, or disc), resulting in SF fibers with various morphologies, indirectly.^63^ With a coaxial electrospinning technique, core‐shell structured SF fibers can be devised by manipulating the composition of the extruded droplets using a dual‐channel injector 64 as illustrated in Figure 4C. These advantages in diverse fiber morphologies, controlled compositions, and desirable nanoscale dimensions enable SF fibers to be widely used in tissue and cell engineering.^65^

### 3.4 Textiles

**FIGURE 5:**
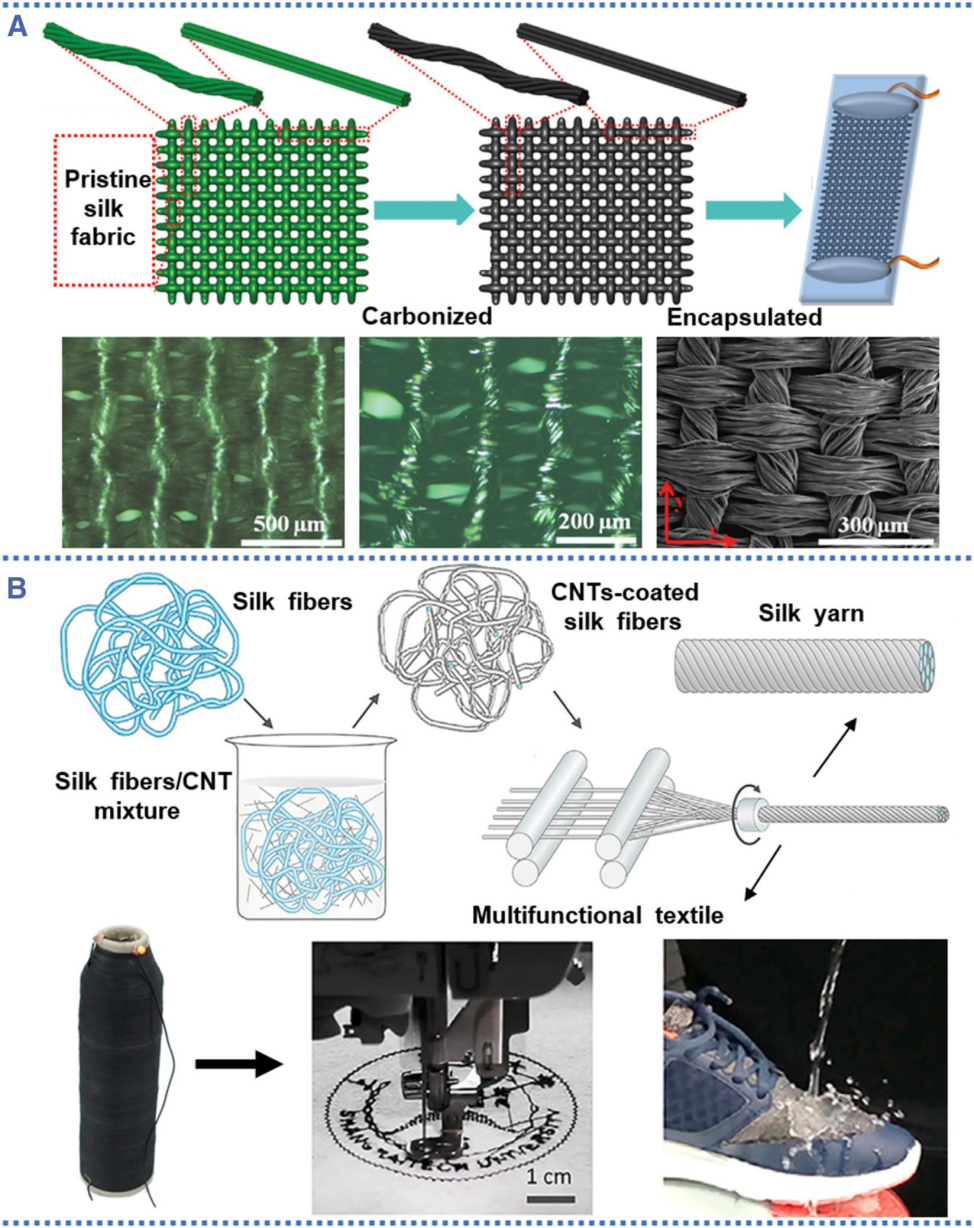
Silk textiles from different treatments. (A) Extremely stretchable silk textiles from pristine silk fabric by using the carbonization technique^28^ (Reprinted with permission. Copyright 2016, John Wiley and Sons). (B) Multifunctional silk textiles form the regenerated silk fibroin by a dip‐coating strategy^71^ (Reprinted with permission. Copyright 2019, Elsevier). The regenerated silk fibroin was obtained from the degummed process and then mixed with carbon nanotubes to prepare multifunctional silk textiles.

Silk fibroin‐based biomedical textiles were widely used as flexible electronics,^66^ general surgery,^67^ tissue engineering,^68^ and esthetic surgery.^69^ Silk textiles are obtained by spinning SF fibers, which have a history dating back thousands of years.^70^ Various natural or RSF fibers with special functions were designed and used to weave biomedical textiles. To meet practical needs, the silk fibroin used for textiles should be modified appropriately, involving structure/composition alterations in situ, and recombinations between molecules. The carbonized technology, as illustrated in Figure 5A, is an effective way to weave smart silk textiles from pure silk fabric.^28^ With high‐temperature carbonization, the natural silk fabric is transformed into nitrogen‐doped carbon nanomaterial with a hierarchical porosity structure and high electrical conductivity. With electrical conductivity and customizable weave structures, these smart silk fibroin textiles are suitable materials for flexible electronics sensors.

However, silk fabrics can also be degummed to produce a RSF solution, which can then be spun into silk fibers and applied to the textile manufacturing process (Figure 5B). Ling et al. reported a functional silk textile with high performance by using partially dissolved silk fiber and carbon nanotubes for e‐textile applications.^71^ In brief, the carbon nanotubes and silk fibers were co‐dissolved in a solution of HFIP, in which the carbon nanotubes were well dispersed and the silk were slightly dissolved on the surface but preserved the main structure of silk fibers. Such a co‐dissolved system allows for the homogeneous assembly of carbon nanotubes on the surface of silk fibers, resulting in carbon nanotube/silk conductive fibers that can be spun into textiles. The resulting multifunctional silk textiles possess high mechanical strength and toughness with solvent resistance, thermal conductivity, and electrical conductivity contributed by carbon nanotubes. This combination of functional materials and spinning/processing technology will result in silk textiles that can satisfy the expanding demands.^72^

## 4 CONDUCTIVE SILK‐BASED MATERIALS

**FIGURE 6:**
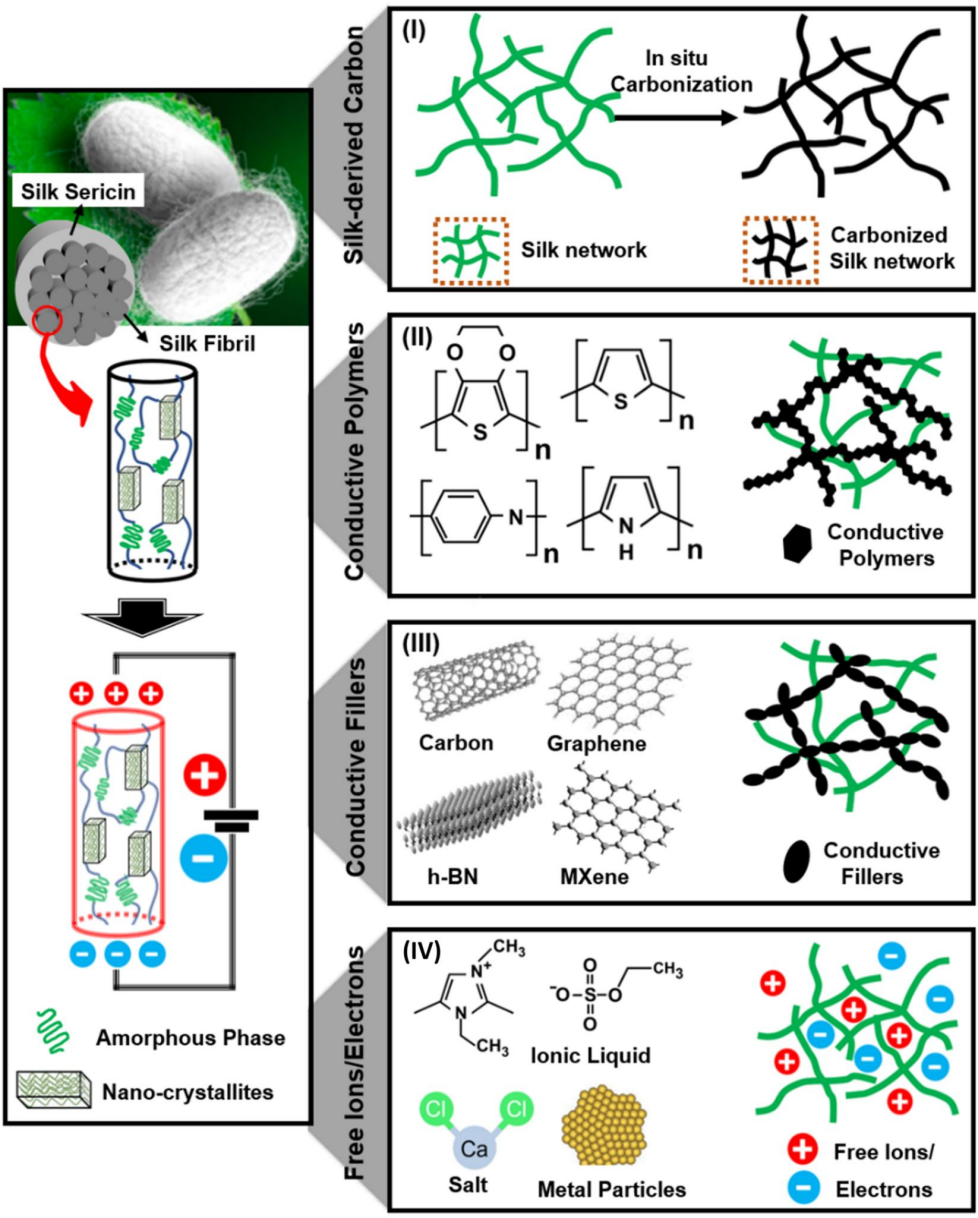
Several structures of conductive silk‐based materials. Left column: Illustration of the conductive silk fibroin with *β*‐crystallite and amorphous phase. Right column: Schematic diagrams of conductive silk‐based materials synthesized by (I) in situ carbonization, (II) conductive polymer composite, (III) conductive fillers infill, and (IV) free charge/ions, respectively.

In general, conductive silk‐based advanced materials are mainly prepared by (1) in situ carbonating the silk polymer networks, (2) polymerizing conductive polymer within silk polymer networks, (3) doping conductive fillers within silk polymer networks, and (4) embedding free charges/ions (such as ionic liquid, salt ion, and metal ion) within silk polymer networks (Figure 6). According to the conducting mechanism, the formed conductive silk‐based materials can be divided into three categories: ionic, electronic, and hybrid ionic‐electronic conductive composite.^73^ By combining different conductive mechanisms and forms (Table 1), the silk‐based conductive materials have been extensively used in various fields, such as biomedical engineering.^83^, ^84^, ^85^, ^86^

**TABLE 1:** Paradigms of multifunctional conductive silk‐based materials in biological interfaces.

Composition	Forms	Processing methods	Applications	Ref
SF/PPy	Film	Chemical polymerization	ECG signals monitoring	74
SF‐PVA/borax	Hydrogel	Physical polymerization	Strain sensing platform	75
SF‐MXene	Hydrogel	Physical polymerization	Bone regeneration	76
SF‐CNT	Textile	Dip‐coating strategy	Electronic yarns	71
SF‐PAM/GO‐PEDOT:PSS	Hydrogel	Physical polymerization	Strain sensing platform	77
SF‐GO/Ca^2+^	Film	Screen printing	Epidermal electronics	24
SF‐Au	Film	Transferred printing	Neural encoding	78
SF‐GMA	Scaffold	3D printing	Tissue scaffold	45
SF‐AgNW	Scaffold	Spinning coating	Ionotronic skin	79
SilkNCT	Textile	Thermoplastic molding	Electrode sensor	23
SF‐PEDOT	Scaffold	Physical polymerization	Nerve regeneration	80
Carbonized SF	Fiber	Thermoplastic molding	Carbon/material electronics	16
SF‐AgNPs	Scaffold	Chemical polymerization	Antibacterial application	81
SF‐HPR	Scaffold	Enzymatic crosslinking	Cartilage regeneration	82
SF‐CNT/Ca^2+^	Fiber	Bioinspired spinning	Sensing platform	62
SF‐Au	Film	Soft assembly	Neural interfacing	25

Abbreviations: AgNPs, Silver nanoparticles; AgNWs, Conducting silver nanowires; CNT, Carbon nanotube; ECG, electrocardiogram; GMA, Glycidyl methacrylate; GO, Graphene Oxide; HRP, Horseradish Peroxidase; PAM, Polyacrylamide; PEDOT:PSS, Poly(3,4‐ethylenedioxythiophene) polystyrene sulfonate; PPy, Polypyrrole; PVA, Polyvinyl alcohol.

## 5 APPLICATIONS

### 5.1 Electronics and electrochemical sensors

**FIGURE 7:**
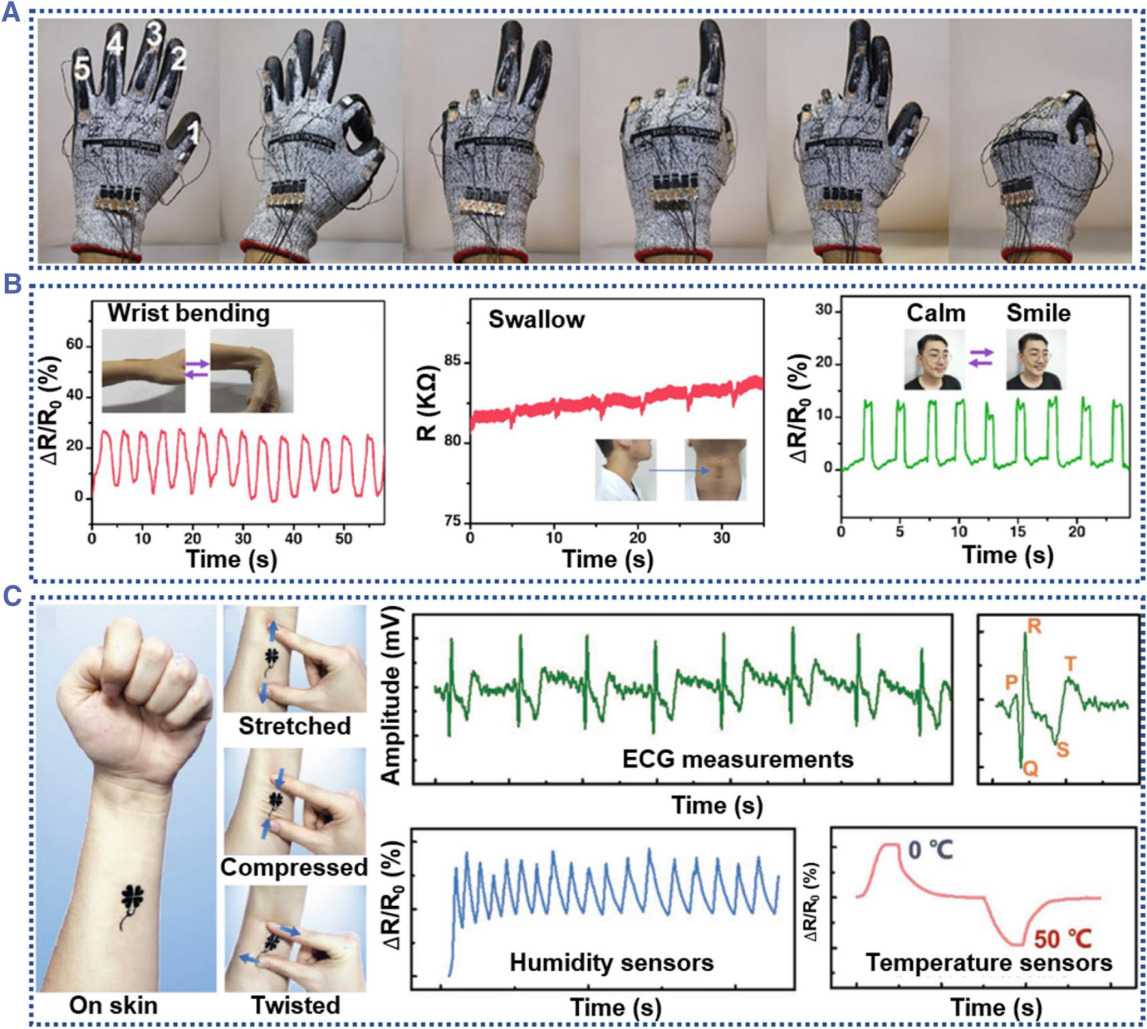
Wearable flexible sensor based on conductive silk‐derived materials. (A) An adhesive, self‐healing, and conductive silk‐graphene hydrogel used for electronic glove (E‐glove) construction. This E‐glove with five sensor channels can distinguish different finger actions^26^ (Reprinted with permission. Copyright 2021, John Wiley and Sons). (B) SF/PVA composite conductive hydrogel strain sensor for tracking physiological signals. This multifunctional hydrogel strain sensor can detect minute alterations in the body, such as light smiling, laughing, breathing quickly, and breathing deeply^92^ (Reprinted with permission. Copyright 2021, American Chemical Society). (C) The Gr/SF/Ca^2+^ hydrogel used for surrounding environment sensing, including temperature variation and humidity^24^ (Reprinted with permission. Copyright 2019, John Wiley and Sons).

In recent years, flexible electronics containing conductive silk fibroin materials are rapidly developing and are widely used in the monitoring of human physiological information.^87^, ^88^, ^89^ For example, conductive SF materials are frequently employed to produce a conventional pressure and a strain sensor.^77^ This sensor can be used to track a variety of motions in the human body, such as body movements, facial expressions, and pulse fluctuations.^90^, ^91^ It converts mechanical motion into an electrical signal based on a change in electrical resistance or capacitance to achieve signal conversion. Because of their intrinsic superiorities, such as high mechanical resilience, good mechanical compliance, and controllable conductivity, flexible electronics based on conductive SF materials can achieve high sensitivity conversion and precise measurement of signals, particularly in wearable epidermis sensors. Alireza Dolatshahi‐Pirouz et al. demonstrated a conductive silk‐graphene hydrogel with strong adhesive, self‐healing, and reconfigurable capabilities for body physiological information sensing.^26^ In brief, the graphene component gave the composite hydrogel significant electrical and thermal conductivity and the hydrogen and electrostatic bonds that formed between silk fibrin, calcium ion, and the graphene component improved the hydrogel's mechanical capabilities. Based on the multifunctional hydrogel, the authors created a five‐channel integrated electronic glove in the hopes that each channel might distinguish different finger actions. And the experimental findings supported their hypotheses (Figure 7A) that the E‐glove was able to recognize many signs through five output channels, including victory, stop, and the numbers. Additionally, Zuo et al. also developed a self‐adhesive, self‐healing, and conductive silk fibroin hydrogel based on the borate ester and hydrogen bonding for flexible strain sensors.^92^ By using such a hydrogel strain sensor, they were able to track physiological signals, such as light smiling, laughing, breathing quickly, and breathing deeply (Figure 7B). Though aiming for high sensing sensitivities, the involved materials should also be stable and responsive to changes in electrical capacitance or resistance. The conductive materials must also possess additional unique qualities, such as high tensile strength, self‐healing, and self‐adhesiveness.^93^

Apart from human motion information sensors, conductive silk materials are also used as functional materials for humidity sensors, temperature sensors, and electrochemical sensors. As shown in Figure 7C, Zhang et al. reported a conductive silk E‐tattoo with numerous stimuli, such as strain, temperature, and humidity. The E‐tattoos are made by screen printing Gr/SF/Ca^2+^ suspension and are soft and highly flexible.^24^ Due to this flexible characteristic, the E‐tattoo can adhere to human skin in a conformal manner and tolerate arbitrary skin deformation without mechanical delamination failure. Taking advantage of the graphene flakes that distributed in the Gr/SF/Ca^2+^ matrix, the author developed an environmentally sensitive multifunctional sensor to strain, temperature, and humidity. All these results suggested that the goal of developing multifunctional conductive SF materials is to develop smart sensors with multiple senses for practical applications.

**FIGURE 8:**
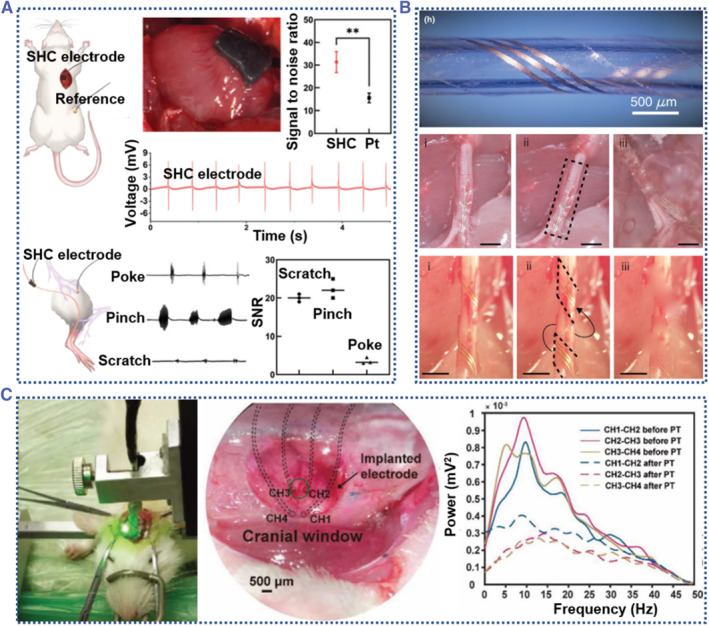
Implantable electronics based on conductive silk‐derived materials. (A) The hydrogel electrode made of SF/CNT for ECG signal recording and sciatic nerve stimulation during in vivo implantation^95^ (Reprinted with permission. Copyright 2022, The Royal Society of Chemistry). (B) An SF/Au electrode pattern film for probing neural–vascular activity^25^ (Reprinted with permission. Copyright 2020, Elsevier). (C) A soft and stable SF film for seamless tissue integration and electronic signal sensing^96^ (Reprinted with permission. Copyright 2021, John Wiley and Sons).

Owing to their excellent biocompatibility, good tissue compliance, extraordinary chemical turnability, and tunable electrical conducting characteristic, conductive silk‐based materials, particularly composites with conjugated polymers,^94^ are of great interest for implantable electronics. By collecting or sending electrical signals, implantable electronics were able to monitor electrophysiological signals, modulate electrogenic tissues, and control the release of biomolecules at the interface between tissues. Thus, conductive silk fibroin materials are of great candidate materials for developing ideal bioelectronic interfaces to treat a variety of chronic ailments. For the treatment of neurological disorders, Fan et al. reported a hydrogel‐based electrode made of functionalized carbon nanotubes (CNTs) and the SF hydrogel.^95^ The CNTs dispersed in the hydrogel network can form a high current permeable network, endowing the hydrogel electrode with high electronic sensitivity. Moreover, the inherent advantages of such a hydrogel electrode are also further increased by the good biocompatibility and mechanical compatibility with living tissues. As a result, in addition to recording ECG signals, these hydrogel electrodes also exert remarkable performance as a neural electrode for sciatic nerve stimulation during in vivo implantation (Figure 8A).

Apart from bulk hydrogel materials, the silk fibroin films with designed electrode arrays are also employed as neural signal monitors. This SF film usually adopts a sandwich structure with a thin layer conductive electrode pattern deposited in two silk layers. As demonstrated by Thakor et al., they created conductive electrode patterns on the silk by depositing a thin layer of Au (Figure 8B).^25^ The formed silk‐based electrode array can be used for rat sciatic nerve recording to obtain a reliable nerve signal conduction velocity, and the result is consistent with nerve conductions.^97^ Chen et al. developed a pattern electrode by using PEGylated‐SF/PEDOT:PSS films (Figure 8C). They employed PEDOT:PSS as a conductive route member and PEG polymer as an SF stabilizer. Of note, all these materials were specifically chosen to avoid the toxic products or metabolites, which will bring poor biocompatibility to the formed electrodes, thereby affecting the bioactivity of the implanted device. Based on the PEGylated‐SF/PEDOT:PSS films, they created a conformable electrode for rat brain implantation and obtained the localized neural signals from the different regions (CH_1_‐CH_4_, CH_2_‐CH_3_, and CH_3_‐CH_4_). However, one of the application obstacles to these implantable SF materials is to create an electrode with desirable qualities like high sensitivity, biocompatibility, and multifunctionality. As a result, the performance of these implantable devices is hindered in the compromisation between integrated functions.

**FIGURE 9:**
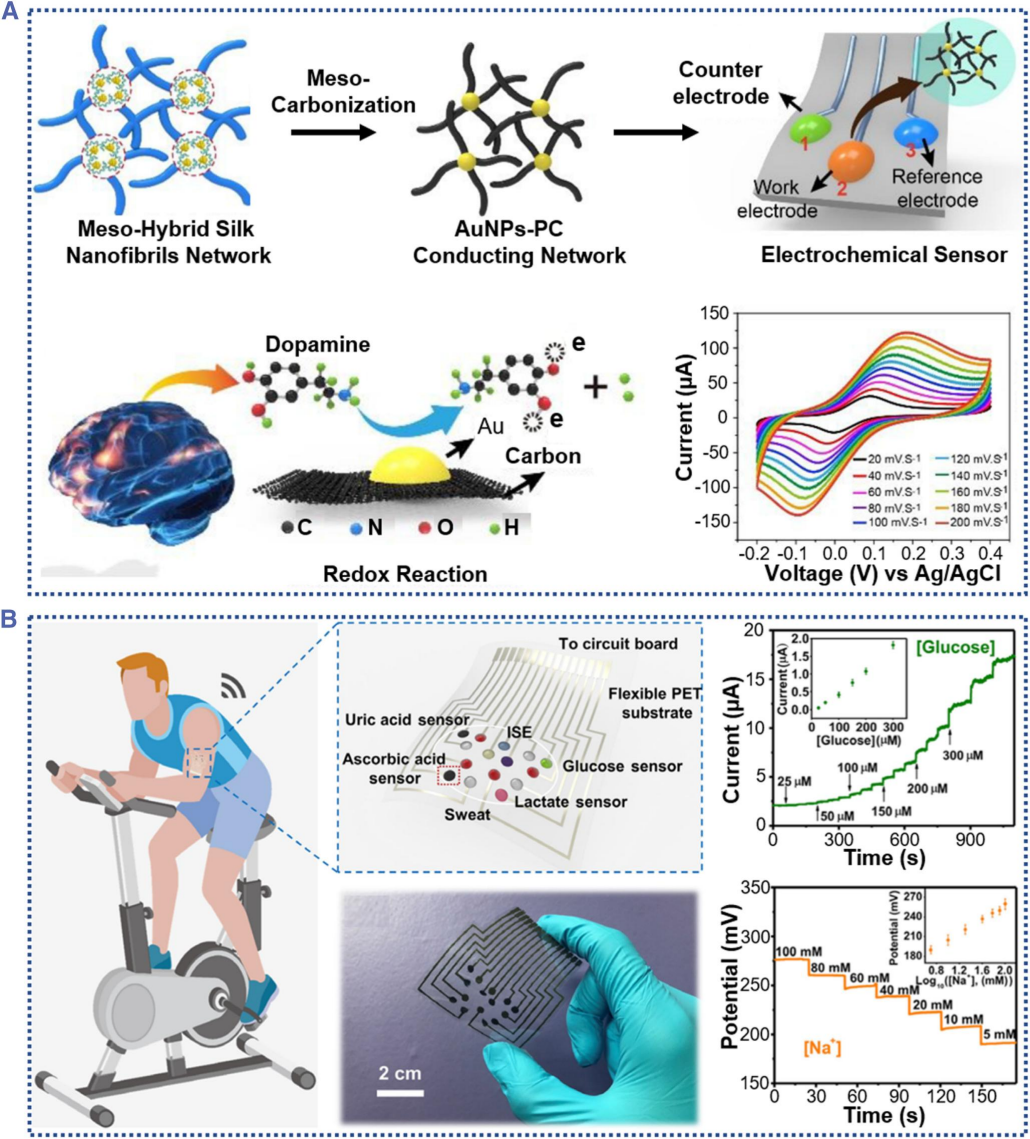
Electrochemical biosensors based on conductive silk‐derived materials. (A) Schematic illustrations of the AuNPs‐PC conducting network obtained by carbonization of SF‐AuNPs hybrid materials. The AuNPs‐PC hybrid can be severed as a working electrode (electrochemical sensor) for the detection of dopamine in the redox reaction^104^ (Reprinted with permission. Copyright 2019, American Chemical Society) (B) An SF‐based carbon textile (SilkNCT) multiple electrochemical sensor array used for wearable sweat analysis patch. The SilkNCT electrochemical sensor can be used to detect various components of sweat^23^ (Reprinted with permission. Copyright 2019, The Authors, published by AAAS).

Biosensors based on silk fibroin and their derivatives also attract great interest due to their ability to detect various small biomolecules, including biochemicals, proteins, electrolytes, and disease‐specific genes.^98^, ^99^ The abundant functional groups in SF polymer chains, together with the complexity of physical interaction between molecules, can serve as excellent targets for the designing of novel detection systems for diverse diagnostic applications.^100^ For example, various enzymes and aptamers can target the SF molecular chain by functional groups amino and hydroxyl, which improve their stability in the detection systems.^101^, ^102^ By utilizing a physical interaction, some functional nanoparticles or fillers can immobilize the SF molecular networks to promote their stability.^103^ Liu et al. developed an electrochemical sensor based on the carbonized silk fibroin/AuNPs (AuNPs‐PC) hybrid materials (Figure 9A).^104^ The formed AuNPs‐PC exhibited good electronic performance and could be employed as a working electrode for the biomolecular (dopamine) sensor by monitoring the electron transfer during the redox reaction. The as‐developed biosensors showed a linear relation between dopamine concentration and peak current intensity, demonstrating excellent electrochemical performance. For this research, the author has revealed that the key to achieving superior electrochemical performance is the carbon material derived from the silk fibroin nanofiber network. In according to this work, Zhang et al. reported an electrochemical sensor array based on the silk‐derived carbon textiles (SilkNCT).^23^ Owing to the hierarchical and porous structure of SilkNCT, the resulting electrode has efficient electron transport and good contact with reactants, which endows the electrode sensor with high sensor sensitivity (Figure 9B). As a result, the authors integrated the SilkNCT‐based material as a working electrode of wearable electrochemical sensor for monitoring multiple biomarkers in sweat, including electrolytes (e.g., Na^+^ and K^+^), biomolecules (e.g., uric acid and ascorbic acid), and metabolites (e.g., glucose and lactate). In this study, the real‐time monitoring data of multiple biomarkers in sweat were compared to data from commercially available assays. The results showed that the integrated SilkNCT‐based sensor had good selectivity, high sensitivity, and long‐term stability for all biomarkers to be detected. All these results demonstrate that the silk‐derived carbon materials could serve as a promising electrode array for multiplex biomarker analysis.^105^, ^106^

### 5.2 Tissue/cell engineering

Conductive silk‐based scaffolds are applied in tissue engineering. Combined with tissues/cells, these scaffolds serve the regeneration of tissues/organs, replacement of damaged sites, and the regulations of cell behaviors.^107^, ^108^, ^109^ At the cellular level, the electrical stimulation on conductive silk scaffold can activate intracellular signaling pathways and change the intracellular microenvironment, thereby affecting cell migration, differentiation, and proliferation.^110^, ^111^, ^112^ Electrical stimulation is one of the most effective techniques of neural stimulation to promote nerve regeneration. Efficient electrical stimulation activity relies heavily on scaffolds with improved electrical conductivity, which facilitates the application of an ionically conductive tissue fluid environment. However, the application of these conductive scaffolds is hindered due to the electrical current used for stimulation that may trigger other healthy tissues and have unintended effects.

**FIGURE 10:**
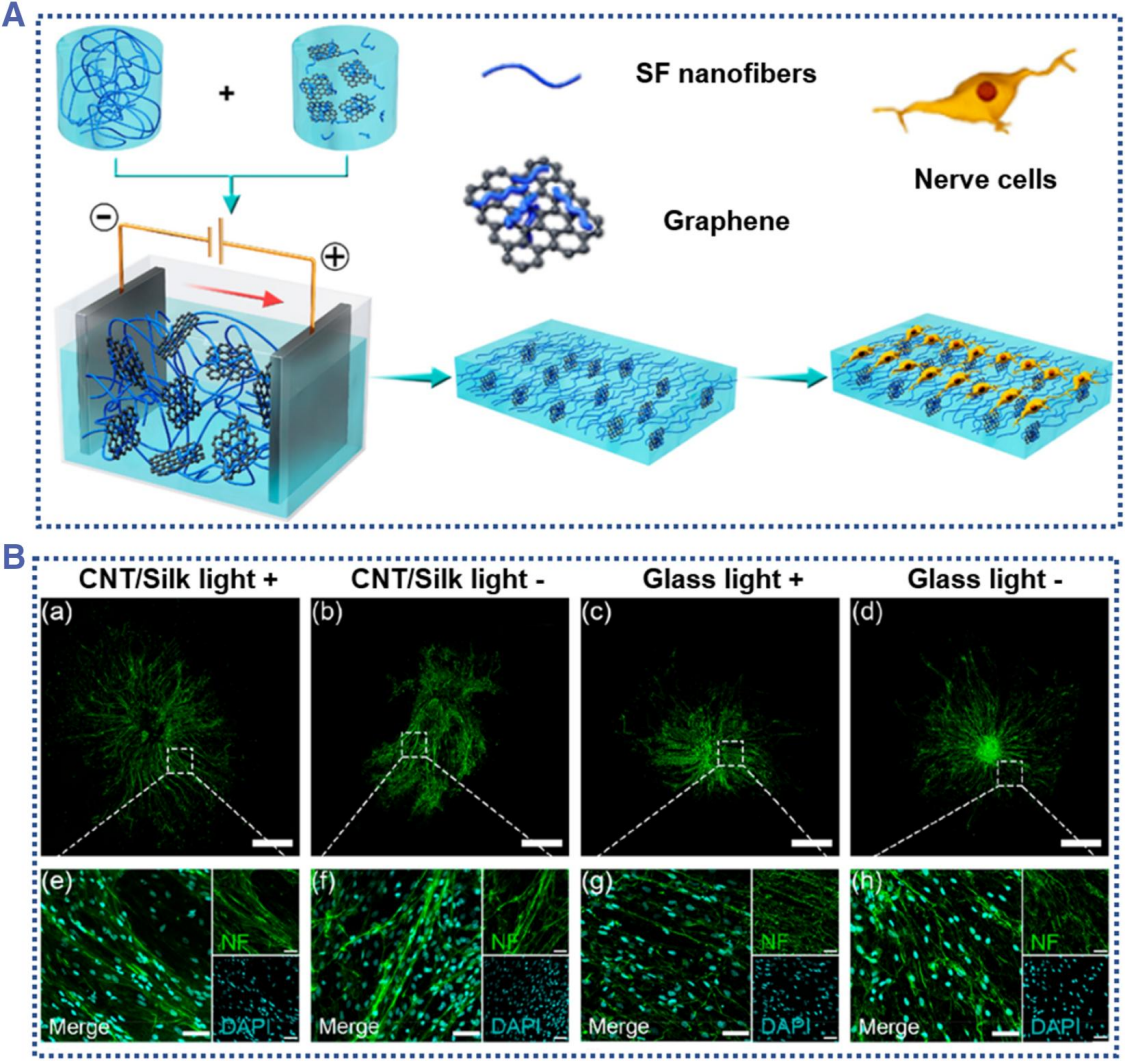
Conductive silk‐derived materials for cell scaffolds. (A) The conductive silk‐graphene hydrogel was used as a nerve cell scaffold to induce cell behaviors^113^ (Reprinted with permission. Copyright 2019, American Chemical Society). (B) The conductive silk‐carbon nanotube scaffold was used to promote neural growth through neural stimulation^114^ (Reprinted with permission. Copyright 2022, American Chemical Society).

The cell behaviors shall be impacted by scaffold components, surface topography, and/or additional stimulating factors. David L. Kaplan et al. developed silk graphene hydrogel for nerve cell behavior regulation.^113^ The authors systematically investigated the synergistic effect of hydrogels with different stiffness, nanofiber structures, and bioactive graphene sheets on neural cell behaviors (Figure 10A). The synergistic effects of numerous signals on the activity of nerve cells are clearly shown by these scaffolds. Based on these results, silk‐based conductive hydrogel scaffolds can be created to investigate nerve regeneration and the relationship between cells and biomaterials. Yang et al. reported a conductive and biocompatible SF scaffold with photoacoustic capabilities for neural stimulation and regeneration.^114^ The SF scaffolds embedded with carbon nanotubes provide ECM‐like environments for tissue growth. The carbon nanotubes of the scaffold absorbed pulsed near‐infrared II (NIR‐II) light and converted it into acoustic energy, which stimulate nerve growth by activating neurons (Figure 10B). Generally, these results demonstrate that the conductive silk‐based scaffold not only promotes nerve adhesion and protects nerve functions, but also possesses programmable mechanical properties, tunable drug loading capacities, and a controllable rate of biodegradation, all of which are essential for neural cell scaffolds.

**FIGURE 11:**
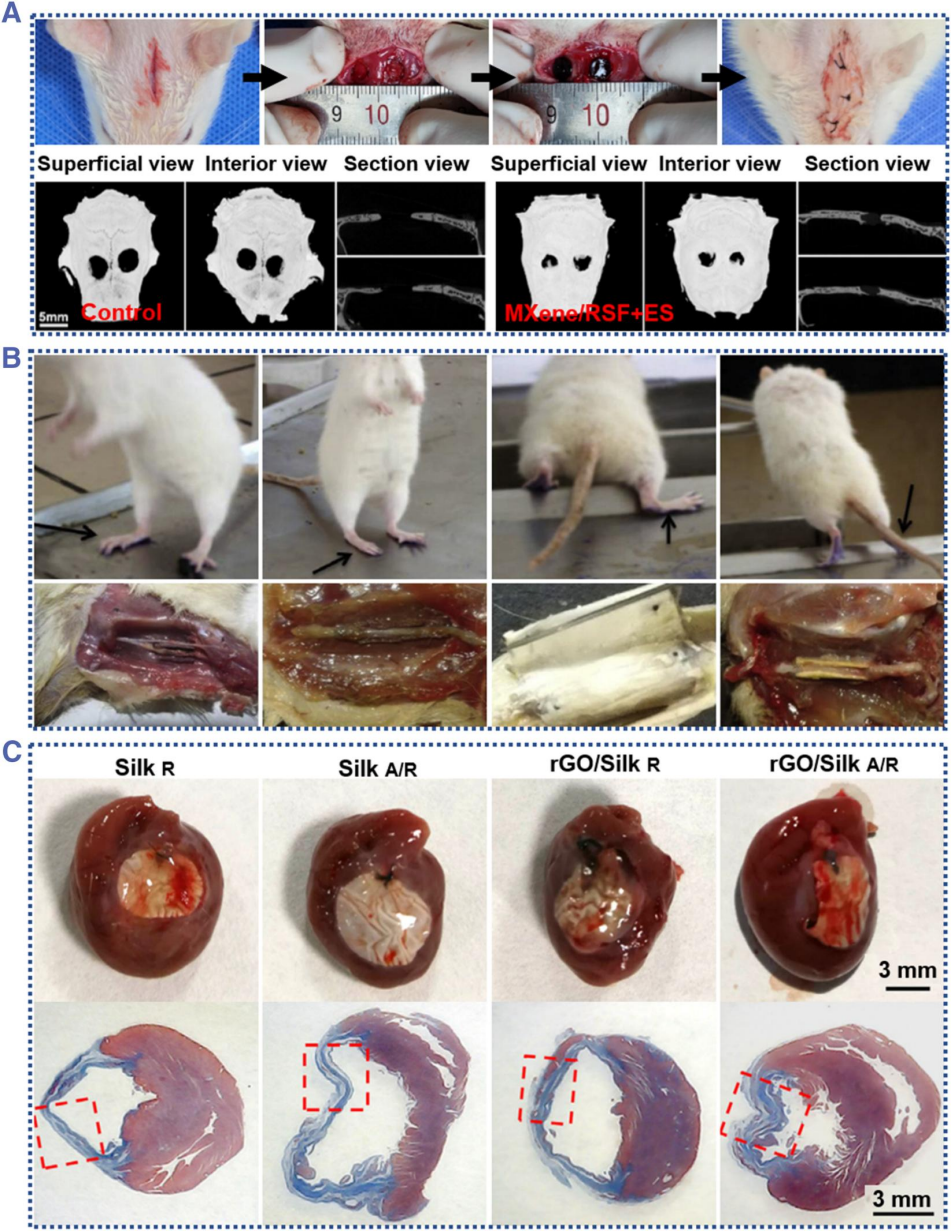
Conductive silk‐derived scaffolds for tissue/bone regeneration. (A) An RSF/MXene nanocomposite hydrogel for bone regeneration^76^ (Reprinted with permission. Copyright 2023, The Authors, published by Elsevier). (B) An RSF/Au nanocomposite conduit for neural regeneration^116^ (Reprinted with permission. Copyright 2015, Elsevier). (C) An RSF/RGO nanocomposite matrice for post‐infarction myocardial function recovery^30^ (Reprinted with permission. Copyright 2022, Elsevier).

The choice of scaffold also affects the rapid and complete regeneration of tissue or organs. To promote the regeneration of tissue and organs, conductive silk‐based scaffolds can be employed as a medium to offer electrical stimulation, an appropriate structure, and a real tissue‐like composition. In the field of bone regeneration, electrical stimulation is widely used to regulate the osteogenic function of osteoblasts because of the bone microenvironment's bioelectric properties.^115^ Jiang et al. reported an RSF/MXene nanocomposite hydrogel for bone regeneration.^76^ In this work, because the SF formed a β‐sheet structure on the surface of MXene nanosheet, the MXene nanosheets can avoid restacking and oxidation and maintain their excellent electrical stimulating capacity. By using the formed RSF/MXene hydrogel as a scaffold for the cranial defect models, the researchers observed enhanced bone regeneration and improved angiogenesis under electrical stimulating in vivo (Figure 11A). These indicate that silk‐based conductive materials are appropriate for creating electrical microenvironments for the regeneration of bone, and they could provide a promising approach for promoting direct osteogenesis and neovascularization.

The use of nerve conduits made of biopolymers is an alternative approach to nerve autograft.^117^, ^118^ SF nanofiber scaffolds have been proven to be a promising biopolymer with biocompatibility and biomimetic characteristics for the fabrication of nerve conduits.^119^ As shown in Figure 11B, Utpal Bora et al. reported the RSF/Au nanocomposite conduits used for neural regeneration.^116^ The resulting RSF/Au conduit was successfully evaluated using the sciatic nerve injury rat models. In comparison to these nerve conduits made of synthetic materials and collagen‐based nerve conduits, the RSF/Au conduit has a certain superior performance. The group of RSF/Au conduit was also able to perform complex locomotory activities like stretching and jumping with an excellent sciatic function index (SFI) and led to a normal life. The experiment group of RSF/Au was able to perform actions, such as jumping and stretching, and continued to live normally. In addition, conductive SF scaffolds can also be used to construct electrical microenvironment suitable for cardiac regenerative medicine. Zhang et al. developed the rGO/SF matrices with anisotropic electrical signal propagation ability and enhanced suturability (Figure 11C).^30^ Such rGO/silk matrices were employed as cardiac patches, demonstrating their potentialities in improving the myocardial function of rat models.

**FIGURE 12:**
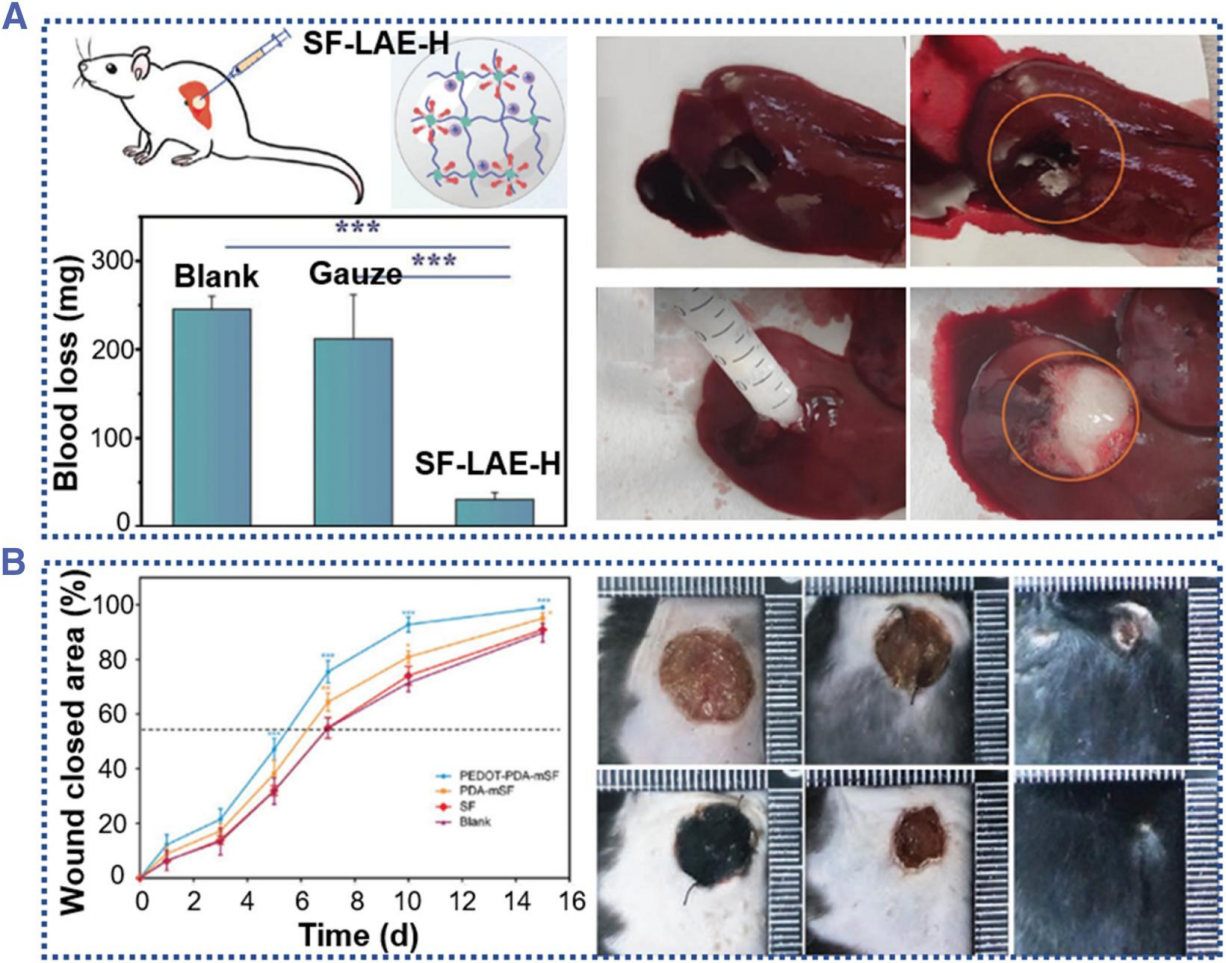
Conductive silk‐derived hydrogels for wound dressing. (A) The SF‐LAE hydrogel used for treating noncompressible hemorrhage wound^32^ (Reprinted with permission. Copyright 2022, John Wiley and Sons). (B) The SF‐PDA‐PEDOT patch used for wound healing and diagnosis in diabetes^120^ (Reprinted with permission. Copyright 2021, John Wiley and Sons).

To date, conductive silk‐based materials have demonstrated their widely applications in other biomedical engineering, such as functional scaffolds for tissue/organ wound recovery and drug delivery systems. Liu et al.^32^ developed ion‐conducting SF hydrogels using ethyl lauroyl arginine hydrochloride (LAE) as a hydrogel initiator (Figure 12A). With the help of cationic surfactant LAE, the formed SF hydrogels have high amphiphilic and antibacterial activity, which are especially beneficial for wound recovery. Wang et al. also developed a functional SF‐PDA‐PEDOT hydrogel with the help of polydopamine (PDA) (Figure 12B). The developed PEDOT‐PDA‐SF hydrogel patch exhibited enhanced mechanical and good conductivity properties and miraculous effects on diabetic wound healing.^120^ Therefore, functional conductive SF materials are expected to pave the way for biointerface materials, thus broadening their biomedical applications.

## 6 CONCLUSION AND OUTLOOK

Silk fibroin, as a natural biopolymer, manifests intrinsic benefits, such as good mechanical flexibility, biocompatibility, and degradability, with flexible and diverse forms in applications. These characteristics are extremely suitable for developing next‐generation biological interfaces. With the help of functional groups on the silk fibroin polymer, various conductive fillers were combined with SF to fabricate conductive materials. Conductive SF materials have been presented in a variety of forms, including hydrogels, fibers, films, and scaffolds by using technologies, such as 3D printing, electrospinning, freeze drying, and soft lithography. Multiforms of conductive SF are used to construct smart biological interfaces, which can play an important role in the fields of sensing and tissue engineering.

Nevertheless, SF‐based conductive materials are also facing many challenges while making great progress. The combination of conductive fillers and silk fibroin polymers is one of the strategies to construct advanced SF materials with conductivity. This strategy often faces obstacles in either the creation of functional materials or the actual application process. Conducive SF materials made from carbon‐related fillers have good electronic conductive but poor biological activity. Taking advantage of conducting polymers endows SF with good biocompatibility and conductivity, but also with poor mechanical strength. And ion‐type SF materials are typically plagued by ion diffusion. In terms of integrating conductive materials with biological tissue, challenges mainly exist in the spillover current and the high interface voltage, leading to the localized heat effect, electrode degradation, and biocompatibility issues, especially when those materials are serving as a scaffold for electrical simulations.

In conclusion, conductive SF materials with high conductivity, good mechanical robustness, and good biocompatible are the basic requirements for their bio interface applications. In some cases, additional functionalities, such as self‐healing and adhesive, are also necessary for practical applications. Therefore, the hot topic of the future for conductive silk materials should be expanding the functionality of materials while maintaining inherent attributes. Apart from material designs, we shall also focus more on gaining a deeper understanding of tissue–biomaterial interactions, which is critical for the development of practical SF materials. We are convinced that conductive SF materials with tailored properties will be able to meet the demands of biological interface, such as flexible, wearable, and implantable microsystems.

## AUTHOR CONTRIBUTIONS

The manuscript was written through contributions of all authors. All authors have given approval to the final version of the manuscript.

## CONFLICT OF INTEREST STATEMENT

The authors declare no conflict of interest.
